# Bis(2-amino­pyridine-κ*N* ^1^)silver(I) nitrate

**DOI:** 10.1107/S1600536808031292

**Published:** 2008-10-04

**Authors:** Hoong-Kun Fun, Jain John, Samuel Robinson Jebas, T. Balasubramanian

**Affiliations:** a X-ray Crystallography Unit, School of Physics, Universiti Sains Malaysia, 11800 USM, Penang, Malaysia; b Department of Physics, National Institute of Technology, Tiruchirappalli 620 015, India

## Abstract

The asymmetric unit of the title compound, [Ag(C_5_H_6_N_2_)_2_]NO_3_, consists of one and a half each of both cations and anions, the other halves being generated by crystallographic inversion centres. One of the Ag^I^ atoms lies on an inversion center and one of the nitrate ions is disordered across an inversion center. Each Ag^I^ atom is bicoordinated in a linear geometry by two N atoms from two 2-amino­pyridine ligands. In the crystal structure, the cations and anions are linked into a two-dimensional network parallel to (001) by N—H⋯O and C—H⋯O hydrogen bonds.

## Related literature

For general background, see: Kristiansson (2000 ▶); Windholz (1976 ▶). For related structures, see: Deng *et al.* (2004 ▶); Yang *et al.*(2004 ▶). For bond-length data, see: Allen *et al.*(1987 ▶); Jebas *et al.* (2007 ▶).
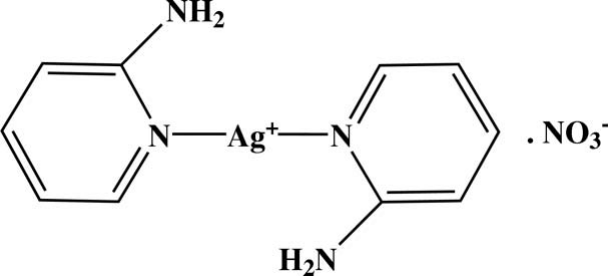


## Experimental

### 

#### Crystal data

[Ag(C_5_H_6_N_2_)_2_]NO_3_
*M* *_r_* = 358.12
Monoclinic, 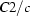

*a* = 43.3371 (10) Å
*b* = 5.8517 (1) Å
*c* = 15.1632 (3) Å
β = 100.502 (1)°
*V* = 3780.91 (13) Å^3^
*Z* = 12
Mo *K*α radiation
μ = 1.61 mm^−1^
*T* = 100.0 (1) K
0.71 × 0.31 × 0.16 mm

#### Data collection

Bruker SMART APEXII CCD area-detector diffractometer
Absorption correction: multi-scan (*SADABS*; Bruker, 2005 ▶) *T*_min_ = 0.394, *T*_max_ = 0.782
42747 measured reflections
9821 independent reflections
8093 reflections with *I* > 2σ(*I*)
*R*_int_ = 0.032

#### Refinement

*R*[*F* ^2^ > 2σ(*F* ^2^)] = 0.035
*wR*(*F* ^2^) = 0.075
*S* = 1.06
9821 reflections
293 parameters
3 restraints
H atoms treated by a mixture of independent and constrained refinement
Δρ_max_ = 0.91 e Å^−3^
Δρ_min_ = −1.23 e Å^−3^

### 

Data collection: *APEX2* (Bruker, 2005 ▶); cell refinement: *SAINT* (Bruker, 2005 ▶); data reduction: *SAINT*; program(s) used to solve structure: *SHELXTL* (Sheldrick, 2008 ▶); program(s) used to refine structure: *SHELXTL*; molecular graphics: *SHELXTL*; software used to prepare material for publication: *SHELXTL* and *PLATON* (Spek, 2003 ▶).

## Supplementary Material

Crystal structure: contains datablocks global, I. DOI: 10.1107/S1600536808031292/ci2682sup1.cif

Structure factors: contains datablocks I. DOI: 10.1107/S1600536808031292/ci2682Isup2.hkl

Additional supplementary materials:  crystallographic information; 3D view; checkCIF report

## Figures and Tables

**Table 1:** Hydrogen-bond geometry (Å, °)

*D*—H⋯*A*	*D*—H	H⋯*A*	*D*⋯*A*	*D*—H⋯*A*
N6—H1N6⋯O2^i^	0.83 (3)	2.22 (3)	3.016 (2)	160 (3)
N4—H1N4⋯O3^ii^	0.82 (3)	2.11 (3)	2.879 (2)	157 (3)
N6—H2N6⋯O3^iii^	0.89 (3)	1.99 (3)	2.873 (2)	169 (2)
N4—H2N4⋯O1^iv^	0.82 (3)	2.12 (3)	2.934 (3)	173 (3)
N2—H1N2⋯O4^v^	0.83 (4)	2.32 (4)	3.102 (3)	159 (3)
N2—H1N2⋯O5^v^	0.83 (4)	2.55 (4)	3.282 (4)	148 (3)
N2—H2N2⋯O5^vi^	0.84 (1)	1.95 (1)	2.767 (4)	161 (3)
N2—H2N2⋯O4	0.85 (4)	2.49 (2)	3.210 (4)	144 (3)
C1—H1*A*⋯O3^ii^	0.93	2.41	3.183 (2)	140
C6—H6*A*⋯O5^vi^	0.93	2.43	3.250 (4)	147
C13—H13*A*⋯O1^iv^	0.93	2.52	3.406 (2)	159

Symmetry codes: (i) 
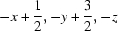
; (ii) 
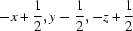
; (iii) 
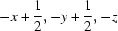
; (iv) 
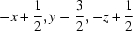
; (v) 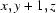; (vi) 
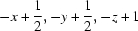
.

**Fig. 1.**
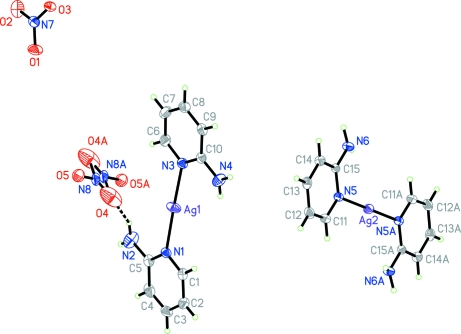
The structure of the title compound, showing 50% probability displacement ellipsoids and the atom numbering scheme. Both components of the disordered nitrate ion are shown.

**Fig. 2.**
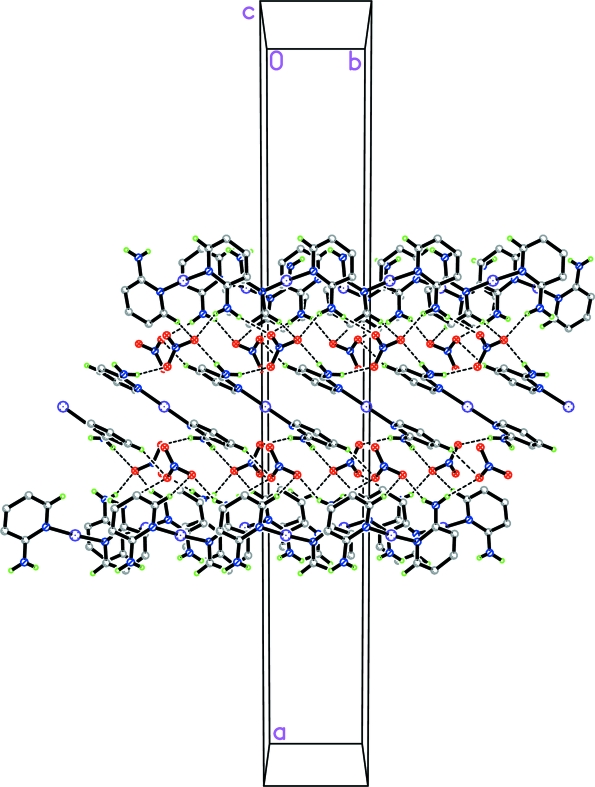
Part of the crystal packing of the title compound, viewed along the c axis. Dashed lines indicate hydrogen bonding.

## supplementary crystallographic information

### Comment

2-Aminopyridine is used in the manufacture of pharmaceuticals, especially antihistaminic drugs (Windholz, 1976). The silver(I) ion exhibits a large flexibility in its coordination with nitrogen-containing aromatic ligands, with coordination numbers ranging from two to eight (Kristiansson, 2000). As a part of our investigation on the binding modes of 2-aminopyridine with the metals, we report here the crystal structure of the title compound.

The asymmetric unit of the title compound consists of one and a half of both [Ag(C_5_H_6_N_2_)_2_]^+^ cation and nitrate anion. The other halves of the cation and anion are generated by crystallographic inversion centres. The Ag2 atom lies on an inversion center. Each Ag^I^ atom is bicoordinated in a linear geometry by two N atoms from two 2-aminopyridine ligands, with an N—Ag—N angle of 175.97 (6)° or 180°. Similar coordination is observed in bis(2-aminopyridine-κ*N*^1^)silver(I) hexafluoroarsenate (Yang *et al.*, 2004) and bis(2-aminopyridine-κ*N*^1^)silver(I) perchlorate (Deng *et al.*, 2004). The bond lengths in 2-aminopyridine ligands are found to have normal values (Jebas *et al.*, 2007; Allen *et al.*, 1987).

In the crystal structure, the cations and anions are linked by N—H···O and C—H···O hydrogen bonds (Table 1), generating a two-dimensional network parallel to the (001) [Fig.2].

### Experimental

2-Aminopyridine in water and silver nitrate in ammonia solution in a molar ratio of 1:1 were mixed with each other and refluxed at 343 K for 6 h. Yellow crystals were obtained after a month on slow evaporation.

### Refinement

The amino H atoms were located in a difference map and allowed to refine freely, with the N2-H1N2 distance restrained to 0.85 Å. The remaining H atoms were positioned geometrically (C—H = 0.93 Å) and refined using a riding model, with *U*_iso_(H) = 1.2*U*_eq_(C). One of the nitrate ion is disordered across an inversion center at (1/4, 1/4, 1/2), with equal occupancy.

### Crystal data

**Table:**

[Ag(C_5_H_6_N_2_)_2_]NO_3_	*F*(000) = 2136
*M**_r_* = 358.12	*D*_x_ = 1.887 Mg m^−^^3^
Monoclinic, *C*2/*c*	Mo *K*α radiation, λ = 0.71073 Å
Hall symbol: -C 2yc	Cell parameters from 9946 reflections
*a* = 43.3371 (10) Å	θ = 2.7–38.4°
*b* = 5.8517 (1) Å	µ = 1.61 mm^−^^1^
*c* = 15.1632 (3) Å	*T* = 100 K
β = 100.502 (1)°	Block, yellow
*V* = 3780.91 (13) Å^3^	0.71 × 0.31 × 0.16 mm
*Z* = 12	

### Data collection

**Table:**

Bruker SMART APEXII CCD area-detector diffractometer	9821 independent reflections
Radiation source: fine-focus sealed tube	8093 reflections with *I* > 2σ(*I*)
graphite	*R*_int_ = 0.032
φ and ω scans	θ_max_ = 37.5°, θ_min_ = 1.0°
Absorption correction: multi-scan (SADABS; Bruker, 2005)	*h* = −74→74
*T*_min_ = 0.394, *T*_max_ = 0.783	*k* = −9→9
42747 measured reflections	*l* = −25→25

### Refinement

**Table:**

Refinement on *F*^2^	Primary atom site location: structure-invariant direct methods
Least-squares matrix: full	Secondary atom site location: difference Fourier map
*R*[*F*^2^ > 2σ(*F*^2^)] = 0.035	Hydrogen site location: inferred from neighbouring sites
*wR*(*F*^2^) = 0.075	H atoms treated by a mixture of independent and constrained refinement
*S* = 1.06	*w* = 1/[σ^2^(*F*_o_^2^) + (0.0208*P*)^2^ + 7.8111*P*] where *P* = (*F*_o_^2^ + 2*F*_c_^2^)/3
9821 reflections	(Δ/σ)_max_ = 0.001
293 parameters	Δρ_max_ = 0.91 e Å^−^^3^
3 restraints	Δρ_min_ = −1.23 e Å^−^^3^

### Special details

**Table:**

Experimental. The data was collected with the Oxford Cyrosystem Cobra low-temperature attachment.
Geometry. All e.s.d.'s (except the e.s.d. in the dihedral angle between two l.s. planes) are estimated using the full covariance matrix. The cell e.s.d.'s are taken into account individually in the estimation of e.s.d.'s in distances, angles and torsion angles; correlations between e.s.d.'s in cell parameters are only used when they are defined by crystal symmetry. An approximate (isotropic) treatment of cell e.s.d.'s is used for estimating e.s.d.'s involving l.s. planes.
Refinement. Refinement of *F*^2^ against ALL reflections. The weighted *R*-factor *wR* and goodness of fit *S* are based on *F*^2^, conventional *R*-factors *R* are based on *F*, with *F* set to zero for negative *F*^2^. The threshold expression of *F*^2^ > σ(*F*^2^) is used only for calculating *R*-factors(gt) *etc*. and is not relevant to the choice of reflections for refinement. *R*-factors based on *F*^2^ are statistically about twice as large as those based on *F*, and *R*- factors based on ALL data will be even larger.

### Fractional atomic coordinates and isotropic or equivalent isotropic displacement parameters (Å^2^)

**Table:**

	*x*	*y*	*z*	*U*_iso_*/*U*_eq_	Occ. (<1)
Ag1	0.167632 (4)	0.28414 (2)	0.419586 (9)	0.03102 (4)	
Ag2	0.0000	0.5000	0.0000	0.02259 (4)	
N1	0.15484 (3)	0.5267 (2)	0.51229 (9)	0.0210 (2)	
N2	0.20046 (5)	0.7366 (4)	0.52126 (17)	0.0409 (5)	
N3	0.17820 (3)	0.0248 (2)	0.32997 (10)	0.0224 (2)	
N4	0.12990 (4)	−0.1493 (3)	0.32282 (14)	0.0315 (3)	
N5	0.02529 (3)	0.2267 (2)	0.06994 (9)	0.0186 (2)	
N6	0.04353 (4)	0.1195 (3)	−0.05863 (9)	0.0219 (2)	
C1	0.12630 (4)	0.5008 (3)	0.53532 (12)	0.0266 (3)	
H1A	0.1152	0.3676	0.5177	0.032*	
C2	0.11291 (5)	0.6585 (4)	0.58280 (13)	0.0342 (4)	
H2A	0.0933	0.6330	0.5978	0.041*	
C3	0.12946 (5)	0.8586 (4)	0.60815 (14)	0.0378 (5)	
H3A	0.1207	0.9715	0.6392	0.045*	
C4	0.15844 (5)	0.8889 (3)	0.58741 (14)	0.0337 (4)	
H4A	0.1697	1.0220	0.6045	0.040*	
C5	0.17137 (4)	0.7160 (3)	0.53960 (12)	0.0245 (3)	
C6	0.20720 (4)	0.0221 (3)	0.30857 (14)	0.0289 (3)	
H6A	0.2203	0.1463	0.3256	0.035*	
C7	0.21823 (5)	−0.1526 (4)	0.26346 (16)	0.0358 (4)	
H7A	0.2382	−0.1466	0.2494	0.043*	
C8	0.19870 (5)	−0.3410 (4)	0.23897 (14)	0.0309 (4)	
H8A	0.2057	−0.4638	0.2089	0.037*	
C9	0.16925 (4)	−0.3435 (3)	0.25950 (12)	0.0247 (3)	
H9A	0.1561	−0.4683	0.2440	0.030*	
C10	0.15902 (4)	−0.1544 (3)	0.30455 (11)	0.0213 (3)	
C11	0.02265 (4)	0.1836 (3)	0.15608 (11)	0.0243 (3)	
H11A	0.0125	0.2908	0.1859	0.029*	
C12	0.03415 (4)	−0.0094 (3)	0.20150 (11)	0.0257 (3)	
H12A	0.0321	−0.0321	0.2608	0.031*	
C13	0.04910 (4)	−0.1712 (3)	0.15613 (12)	0.0241 (3)	
H13A	0.0569	−0.3054	0.1848	0.029*	
C14	0.05227 (4)	−0.1310 (3)	0.06904 (11)	0.0211 (3)	
H14A	0.0621	−0.2379	0.0381	0.025*	
C15	0.04047 (3)	0.0740 (3)	0.02689 (10)	0.0168 (2)	
N7	0.42167 (4)	0.8502 (3)	0.16126 (10)	0.0247 (3)	
O1	0.40629 (4)	0.9276 (3)	0.21680 (11)	0.0424 (4)	
O2	0.44689 (4)	0.9364 (3)	0.15093 (11)	0.0398 (4)	
O3	0.41119 (3)	0.6776 (2)	0.11617 (9)	0.0267 (2)	
N8	0.25139 (8)	0.2036 (5)	0.5180 (2)	0.0269 (6)	0.50
O4	0.22809 (7)	0.2267 (5)	0.52820 (18)	0.0835 (9)	
O5	0.26419 (7)	0.0502 (5)	0.5660 (2)	0.0338 (6)	0.50
H1N6	0.0430 (6)	0.253 (5)	−0.0764 (19)	0.034 (7)*	
H1N4	0.1229 (7)	−0.034 (6)	0.343 (2)	0.045 (8)*	
H2N6	0.0566 (6)	0.034 (4)	−0.0839 (17)	0.028 (6)*	
H2N4	0.1184 (7)	−0.260 (5)	0.310 (2)	0.042 (8)*	
H1N2	0.2107 (8)	0.854 (6)	0.536 (2)	0.061 (10)*	
H2N2	0.2088 (7)	0.625 (4)	0.499 (2)	0.049 (9)*	

### Atomic displacement parameters (Å^2^)

**Table:**

	*U*^11^	*U*^22^	*U*^33^	*U*^12^	*U*^13^	*U*^23^
Ag1	0.05014 (9)	0.01987 (6)	0.02380 (6)	0.00820 (6)	0.00876 (6)	−0.00160 (5)
Ag2	0.02482 (7)	0.02088 (8)	0.02184 (7)	0.00686 (6)	0.00365 (6)	−0.00096 (6)
N1	0.0244 (6)	0.0182 (6)	0.0196 (5)	0.0011 (5)	0.0020 (5)	−0.0018 (4)
N2	0.0327 (9)	0.0262 (8)	0.0647 (14)	−0.0056 (7)	0.0116 (9)	0.0090 (9)
N3	0.0251 (6)	0.0194 (6)	0.0220 (6)	0.0003 (5)	0.0024 (5)	−0.0025 (5)
N4	0.0265 (7)	0.0261 (7)	0.0442 (10)	−0.0002 (6)	0.0124 (7)	0.0056 (7)
N5	0.0197 (5)	0.0196 (6)	0.0166 (5)	0.0014 (5)	0.0037 (4)	−0.0017 (4)
N6	0.0282 (6)	0.0204 (6)	0.0183 (5)	0.0042 (5)	0.0081 (5)	0.0010 (5)
C1	0.0225 (7)	0.0304 (8)	0.0248 (7)	0.0002 (7)	−0.0009 (6)	−0.0001 (6)
C2	0.0263 (8)	0.0494 (12)	0.0262 (8)	0.0118 (8)	0.0030 (6)	0.0016 (8)
C3	0.0428 (11)	0.0406 (11)	0.0260 (8)	0.0212 (9)	−0.0039 (8)	−0.0110 (8)
C4	0.0417 (10)	0.0204 (8)	0.0329 (9)	0.0063 (7)	−0.0095 (8)	−0.0080 (7)
C5	0.0275 (7)	0.0171 (6)	0.0268 (7)	0.0005 (6)	−0.0005 (6)	0.0011 (6)
C6	0.0232 (7)	0.0269 (8)	0.0353 (9)	−0.0029 (6)	0.0019 (6)	−0.0072 (7)
C7	0.0227 (8)	0.0390 (11)	0.0457 (11)	0.0027 (8)	0.0058 (7)	−0.0105 (9)
C8	0.0331 (9)	0.0270 (8)	0.0310 (9)	0.0073 (7)	0.0016 (7)	−0.0077 (7)
C9	0.0299 (8)	0.0179 (6)	0.0236 (7)	0.0009 (6)	−0.0025 (6)	−0.0004 (6)
C10	0.0238 (7)	0.0186 (6)	0.0205 (6)	0.0012 (6)	0.0015 (5)	0.0028 (5)
C11	0.0243 (7)	0.0312 (8)	0.0178 (6)	0.0029 (6)	0.0052 (5)	−0.0023 (6)
C12	0.0255 (7)	0.0350 (9)	0.0161 (6)	−0.0027 (7)	0.0023 (5)	0.0034 (6)
C13	0.0223 (7)	0.0257 (8)	0.0227 (7)	−0.0014 (6)	−0.0002 (5)	0.0064 (6)
C14	0.0226 (6)	0.0185 (6)	0.0224 (7)	0.0012 (5)	0.0044 (5)	0.0015 (5)
C15	0.0170 (5)	0.0169 (6)	0.0164 (5)	−0.0009 (5)	0.0024 (4)	−0.0017 (5)
N7	0.0315 (7)	0.0195 (6)	0.0218 (6)	0.0061 (5)	0.0011 (5)	0.0018 (5)
O1	0.0510 (9)	0.0404 (8)	0.0358 (8)	0.0172 (8)	0.0082 (7)	−0.0138 (7)
O2	0.0404 (8)	0.0332 (8)	0.0431 (8)	−0.0102 (7)	0.0008 (6)	0.0149 (7)
O3	0.0302 (6)	0.0249 (6)	0.0254 (6)	0.0021 (5)	0.0061 (5)	−0.0067 (5)
N8	0.0210 (12)	0.0245 (17)	0.0309 (17)	0.0005 (12)	−0.0069 (12)	−0.0071 (11)
O4	0.106 (2)	0.0855 (18)	0.0630 (15)	−0.0602 (17)	0.0273 (14)	−0.0217 (13)
O5	0.0347 (14)	0.0324 (14)	0.0329 (14)	0.0093 (12)	0.0026 (11)	0.0020 (12)

### Geometric parameters (Å, °)

**Table:**

Ag1—N1	2.1406 (14)	C4—H4A	0.93
Ag1—N3	2.1413 (14)	C6—C7	1.364 (3)
Ag2—N5^i^	2.1115 (14)	C6—H6A	0.93
Ag2—N5	2.1115 (14)	C7—C8	1.398 (3)
N1—C5	1.343 (2)	C7—H7A	0.93
N1—C1	1.354 (2)	C8—C9	1.368 (3)
N2—C5	1.345 (3)	C8—H8A	0.93
N2—H1N2	0.83 (4)	C9—C10	1.414 (2)
N2—H2N2	0.85 (4)	C9—H9A	0.93
N3—C10	1.350 (2)	C11—C12	1.369 (3)
N3—C6	1.354 (2)	C11—H11A	0.93
N4—C10	1.341 (2)	C12—C13	1.397 (3)
N4—H1N4	0.82 (3)	C12—H12A	0.93
N4—H2N4	0.82 (3)	C13—C14	1.373 (2)
N5—C15	1.3473 (19)	C13—H13A	0.93
N5—C11	1.355 (2)	C14—C15	1.411 (2)
N6—C15	1.354 (2)	C14—H14A	0.93
N6—H1N6	0.83 (3)	N7—O2	1.239 (2)
N6—H2N6	0.89 (3)	N7—O1	1.250 (2)
C1—C2	1.363 (3)	N7—O3	1.257 (2)
C1—H1A	0.93	N8—N8^ii^	0.764 (6)
C2—C3	1.390 (4)	N8—O4	1.058 (4)
C2—H2A	0.93	N8—O5	1.223 (4)
C3—C4	1.361 (3)	N8—O4^ii^	1.294 (5)
C3—H3A	0.93	O4—N8^ii^	1.294 (5)
C4—C5	1.418 (3)		
			
N1—Ag1—N3	175.97 (6)	N3—C6—C7	123.67 (17)
N5^i^—Ag2—N5	180.00 (10)	N3—C6—H6A	118.2
C5—N1—C1	118.27 (15)	C7—C6—H6A	118.2
C5—N1—Ag1	124.21 (12)	C6—C7—C8	118.27 (18)
C1—N1—Ag1	116.97 (12)	C6—C7—H7A	120.9
C5—N2—H1N2	120 (2)	C8—C7—H7A	120.9
C5—N2—H2N2	120 (2)	C9—C8—C7	119.52 (17)
H1N2—N2—H2N2	120 (3)	C9—C8—H8A	120.2
C10—N3—C6	118.18 (15)	C7—C8—H8A	120.2
C10—N3—Ag1	122.67 (11)	C8—C9—C10	119.31 (17)
C6—N3—Ag1	118.41 (12)	C8—C9—H9A	120.3
C10—N4—H1N4	121 (2)	C10—C9—H9A	120.3
C10—N4—H2N4	119 (2)	N4—C10—N3	118.52 (16)
H1N4—N4—H2N4	119 (3)	N4—C10—C9	120.47 (17)
C15—N5—C11	118.54 (14)	N3—C10—C9	121.01 (16)
C15—N5—Ag2	120.92 (10)	N5—C11—C12	123.47 (16)
C11—N5—Ag2	119.85 (11)	N5—C11—H11A	118.3
C15—N6—H1N6	119.7 (19)	C12—C11—H11A	118.3
C15—N6—H2N6	118.9 (16)	C11—C12—C13	118.07 (15)
H1N6—N6—H2N6	112 (2)	C11—C12—H12A	121.0
N1—C1—C2	123.88 (19)	C13—C12—H12A	121.0
N1—C1—H1A	118.1	C14—C13—C12	119.62 (16)
C2—C1—H1A	118.1	C14—C13—H13A	120.2
C1—C2—C3	117.93 (19)	C12—C13—H13A	120.2
C1—C2—H2A	121.0	C13—C14—C15	119.34 (15)
C3—C2—H2A	121.0	C13—C14—H14A	120.3
C4—C3—C2	119.80 (18)	C15—C14—H14A	120.3
C4—C3—H3A	120.1	N5—C15—N6	118.38 (14)
C2—C3—H3A	120.1	N5—C15—C14	120.90 (14)
C3—C4—C5	119.50 (19)	N6—C15—C14	120.71 (14)
C3—C4—H4A	120.2	O2—N7—O1	121.89 (18)
C5—C4—H4A	120.2	O2—N7—O3	119.84 (16)
N1—C5—N2	118.52 (18)	O1—N7—O3	118.26 (17)
N1—C5—C4	120.53 (17)	O4—N8—O5	110.4 (4)
N2—C5—C4	120.94 (19)		
			
C5—N1—C1—C2	1.7 (3)	C6—N3—C10—C9	2.1 (2)
Ag1—N1—C1—C2	−170.08 (15)	Ag1—N3—C10—C9	−167.90 (12)
N1—C1—C2—C3	0.7 (3)	C8—C9—C10—N4	177.81 (18)
C1—C2—C3—C4	−1.7 (3)	C8—C9—C10—N3	−2.1 (3)
C2—C3—C4—C5	0.4 (3)	C15—N5—C11—C12	−1.1 (3)
C1—N1—C5—N2	176.31 (18)	Ag2—N5—C11—C12	169.43 (14)
Ag1—N1—C5—N2	−12.5 (2)	N5—C11—C12—C13	−0.7 (3)
C1—N1—C5—C4	−3.1 (3)	C11—C12—C13—C14	1.1 (3)
Ag1—N1—C5—C4	168.05 (13)	C12—C13—C14—C15	0.3 (2)
C3—C4—C5—N1	2.1 (3)	C11—N5—C15—N6	−178.89 (15)
C3—C4—C5—N2	−177.3 (2)	Ag2—N5—C15—N6	10.64 (19)
C10—N3—C6—C7	−0.6 (3)	C11—N5—C15—C14	2.6 (2)
Ag1—N3—C6—C7	169.84 (18)	Ag2—N5—C15—C14	−167.88 (11)
N3—C6—C7—C8	−0.9 (3)	C13—C14—C15—N5	−2.2 (2)
C6—C7—C8—C9	0.9 (3)	C13—C14—C15—N6	179.32 (15)
C7—C8—C9—C10	0.5 (3)	O5—N8—O4—N8^ii^	−177.8 (7)
C6—N3—C10—N4	−177.77 (17)	O4^ii^—N8—O4—N8^ii^	0.0
Ag1—N3—C10—N4	12.2 (2)		

Symmetry codes: (i) −*x*, −*y*+1, −*z*; (ii) −*x*+1/2, −*y*+1/2, −*z*+1.

### Hydrogen-bond geometry (Å, °)

**Table:**

*D*—H···*A*	*D*—H	H···*A*	*D*···*A*	*D*—H···*A*
N6—H1N6···O2^iii^	0.83 (3)	2.22 (3)	3.016 (2)	160 (3)
N4—H1N4···O3^iv^	0.82 (3)	2.11 (3)	2.879 (2)	157 (3)
N6—H2N6···O3^v^	0.89 (3)	1.99 (3)	2.873 (2)	169 (2)
N4—H2N4···O1^vi^	0.82 (3)	2.12 (3)	2.934 (3)	173 (3)
N2—H1N2···O4^vii^	0.83 (4)	2.32 (4)	3.102 (3)	159 (3)
N2—H1N2···O5^vii^	0.83 (4)	2.55 (4)	3.282 (4)	148 (3)
N2—H2N2···O5^ii^	0.84 (1)	1.95 (1)	2.767 (4)	161 (3)
N2—H2N2···O4	0.85 (4)	2.49 (2)	3.210 (4)	144 (3)
C1—H1A···O3^iv^	0.93	2.41	3.183 (2)	140
C6—H6A···O5^ii^	0.93	2.43	3.250 (4)	147
C13—H13A···O1^vi^	0.93	2.52	3.406 (2)	159

Symmetry codes: (iii) −*x*+1/2, −*y*+3/2, −*z*; (iv) −*x*+1/2, *y*−1/2, −*z*+1/2; (v) −*x*+1/2, −*y*+1/2, −*z*; (vi) −*x*+1/2, *y*−3/2, −*z*+1/2; (vii) *x*, *y*+1, *z*; (ii) −*x*+1/2, −*y*+1/2, −*z*+1.
